# Evidence of Negative Capacitance and Capacitance Modulation by Light and Mechanical Stimuli in Pt/ZnO/Pt Schottky Junctions

**DOI:** 10.3390/s21062253

**Published:** 2021-03-23

**Authors:** Raoul Joly, Stéphanie Girod, Noureddine Adjeroud, Patrick Grysan, Jérôme Polesel-Maris

**Affiliations:** 1 Luxembourg Institute of Science and Technology, L-4422 Belvaux, Luxembourg; raoul.joly@list.lu (R.J.); stephanie.girod@list.lu (S.G.); noureddine.adjeroud@list.lu (N.A.); patrick.grysan@list.lu (P.G.); 2 Limpertsberg Campus, University of Luxembourg, 162a Avenue de la Faïencerie, L-1511 Luxembourg, Luxembourg

**Keywords:** zinc oxide, negative capacitance, capacitance modulation, strain sensor, Schottky junction

## Abstract

We report on the evidence of negative capacitance values in a system consisting of metal-semiconductor-metal (MSM) structures, with Schottky junctions made of zinc oxide thin films deposited by Atomic Layer Deposition (ALD) on top of platinum interdigitated electrodes (IDE). The MSM structures were studied over a wide frequency range, between 20 Hz and 1 MHz. Light and mechanical strain applied to the device modulate positive or negative capacitance and conductance characteristics by tuning the flow of electrons involved in the conduction mechanisms. A complete study was carried out by measuring the capacitance and conductance characteristics under the influence of both dark and light conditions, over an extended range of applied bias voltage and frequency. An impact-loss process linked to the injection of hot electrons at the interface trap states of the metal-semiconductor junction is proposed to be at the origin of the apparition of the negative capacitance values. These negative values are preceded by a local increase of the capacitance associated with the accumulation of trapped electrons at the interface trap states. Thus, we propose a simple device where the capacitance values can be modulated over a wide frequency range via the action of light and strain, while using cleanroom-compatible materials for fabrication. These results open up new perspectives and applications for the miniaturization of highly sensitive and low power consumption environmental sensors, as well as for broadband impedance matching in radio frequency applications.

## 1. Introduction

Despite being observed experimentally for several years [1,2,3], negative capacitance (NC) phenomena recently aroused substantial interest due to the growing demand for miniaturized electronic devices and applications. Negative capacitance has already been widely studied in ferroelectric-dielectric structures [4,5,6,7] to improve the subthreshold swing and internal voltage amplification of transistors [8,9]. Moreover, achieving negative capacitance for inductors over a large frequency range would constitute a breakthrough for broadband impedance matching in radio frequency applications [10]. Therefore, planar and easy to process capacitors with negative capacitance values could be used to replace bulky inductors in such applications [11]. The presence of NC phenomena is often accompanied by an increase in capacitance above the conventional geometrical values, localized in a peak preceding the NC values at low frequencies [12,13]. Recent works have reported a significant increase in capacitance in the presence of a light source, where light-generated carriers, e.g., photoexcited electrons, contribute to the conduction mechanisms [14,15,16], while further increasing the peak of positive capacitance values preceding the negative capacitance values [17].

Within this scope, a wide range of materials and structures were investigated to further develop the current understanding of NC-associated phenomena. Among them, semiconductor devices, including Schottky diodes [12,18], p-n junctions [19], heterojunctions [20] or metal-insulator-semiconductor structures [21], are well-known for exhibiting such behaviour [22]. Schottky junctions are one of the simplest rectifying systems, which can easily be processed between a metal and a semiconductor. NC is typically observed when a Schottky diode is forward biased at low frequencies, but its exact origin is still a matter of discussion [23]. An additional metallic contact is needed to integrate Schottky junctions into electronic devices, thus forming a metal-semiconductor-metal (MSM) system. This additional contact can either be ohmic or rectifying, the latter leading to the formation of two back-to-back Schottky diodes in the MSM structure. A common cause of NC in MSM diodes is related to the high-level injection of minority carriers from the forward biased metal-semiconductor (MS) junction into the bulk of the semiconductor for intimate Schottky contacts [24]. Another explanation is attributed to an impact-loss process, with a mechanism similar to the impact ionization process. As the electric field is increased and the interface trap states are filled up, electrons acquire excess energy and collide with electrons trapped at the interface states below the Fermi level [1] at the metal-semiconductor junction, moving them into the metal electrode. The interface states can thus play a major role in the obtention of NC in MS systems and typically present dispersive behaviour depending on their ability to follow the frequency of the applied AC driving voltage [12,18,25].

Among semiconducting materials, zinc oxide (ZnO) has attracted considerable attention in the field of semiconductor devices due to several of its features, such as its material compatibility with cleanroom facilities, low temperature processing or its amenability to wet chemical etching [26]. The resulting physical and chemical properties of the material make it viable and appealing for micro and nanotechnology applications, including the fields of biomedical, energy, sensors and optics [27]. The intrinsic n-type electrical behaviour of ZnO is usually linked to the presence of native point defects [26], such as zinc interstitials and oxygen vacancies, with the latter being the most frequently cited reason [28]. The formation of a Schottky junction between ZnO and a high work function metal leads to the generation of localized interface states at the MS junction, at energies corresponding to point defects native to the bulk semiconductor [29]. Oxygen vacancies have been shown to play a major role at the MS interface by pinning the Fermi level close to their defect level, which increases their density at the interface and makes the junction almost insensitive to the Schottky barrier height [30]. Furthermore, negative capacitance phenomena have been linked to the presence, accumulation and migration of oxygen vacancies at the MS interface in various structures [31,32,33,34]. Oxygen vacancies have also been shown to be responsible for the observation of memristive behaviour in ZnO thin films [35]. However, few articles report on the observation of negative capacitance with ZnO-based materials or structures [32,36,37]. The observation of NC phenomena in these works is limited to low frequency domains (i.e., below 1 kHz), while the impact of a light source on the capacitive properties has not been considered. Moreover, the investigation of NC phenomena with ZnO thin films grown by Atomic Layer Deposition (ALD) is not present in the literature.

In the present work, we report on the evidence of negative capacitance values in a system consisting of piezotronic strain microsensors, where Pt/ZnO/Pt Schottky junctions are integrated at the clamped area of millimetre-sized polyimide cantilevers [38]. The Schottky junctions are made of zinc oxide thin films deposited by ALD on top of a polyimide substrate with bottom platinum interdigitated electrodes (IDE). A complete study was carried out by measuring the capacitance and conductance characteristics under the influence of both dark and light conditions, over an extended range of applied bias voltage and frequency. We report on the observation of negative capacitance values over a wide frequency range, studied between 20 Hz and 1 MHz. The negative capacitance values are preceded by a local increase in the capacitance of the device, which is further marked in the presence of a light source. Additionally, we propose a modulation via the mechanical strain of the capacitive response of the sensors by imposing controlled compressive strain steps to the polyimide cantilevers.

## 2. Materials and Methods

### 2.1. ZnO Thin Films and Pt Metal Electrodes Deposition

The ZnO thin films were synthesized by ALD using precursors of diethylzinc [DEZ, Zn(C_2_H_5_)_2_] (Strem Chemicals, Inc., Bischheim, France) and deionized (DI) MilliQ water (resistivity of 18.2 MΩ.cm at 25 °C), in a commercial ALD reactor (TFS-200, Beneq, Espoo, Finland) with a thermal configuration. On the one hand, the supporting surfaces for the ZnO thin film deposition consisted of 1 × 1 cm^2^ pieces of pristine single crystal Si(100) wafer (grade Monitor, Siegert GmbH, Aachen, Germany), some of them coated with a 200 nm thick Platinum (Pt) layer (deposited by Electron Beam Metal Evaporation) for comparative thicknesses and structural measurements with the polymeric substrates. On the other hand, 75 µm thick polyimide films (Kapton ^®^ HN, DuPont de Nemours, Mechelen, Belgium) were used for the subsequent processing of piezotronic strain microsensors. The substrates were cleaned in acetone, isopropanol and DI water, followed by dehydration for 30 min at 200 °C (ramp: 150 °C/h) prior to the ALD growth of ZnO films. Before being introduced into the ALD reactor, an additional plasma cleaning (Plasma Therm 790 RIE, 50 W, 5 min, in Ar:O_2_ gases environment at 60 mTorr) was performed on the substrates. Alphagaz 2 Argon gas was used for the purging steps during the ALD process, with a global purity ≥99.9999% mol and less than 0.5 H_2_O ppm.mol impurity. The ZnO thin films were elaborated at a substrate temperature varying between 60 °C and 100 °C for structural and electrical characterization, while the deposition of ZnO thin films integrated in MSM Schottky junctions was carried out for a substrate temperature of 80 °C. The following sequence of four steps was used: DEZ pulse (0.1 s), Ar purge (6 s), DI water pulse (0.1 s) and Ar purge (6 s). Thermocouples located inside the ALD reaction chamber are controlling and monitoring the reactor and substrate temperatures to the desired value. The depositions were performed under a constant pressure of 2 mbar, controlled by pressure gauges. A number of loops between 1000 and 2000 was set based on the growth rate of the created ZnO thin films at the different temperatures, in order for the ZnO thin films to obtain a thickness ranging between 150 nm for the structural characterization and 300 nm for the piezotronic strain sensors. A higher thickness of the ZnO thin films contributed to an increased electrical stability response of the Schottky junctions by limiting the apparition of memristive phenomena [39].

Prior to thin film metal deposition, used also as supporting conductive bottom layer to fabricate the interdigitated (IDE) electrodes by lithography and lift-off process [38], the polyimide substrates were exposed to a plasma treatment (60 mTorr, O_2_—38 sccm, Ar—2 sccm, 50 W, 3 min) to improve the adhesion of metal to polyimide [40]. The electrodes and contact pad layers of titanium (5 nm)/platinum (200 nm) were evaporated by Electron Beam Metal Evaporation. The metal evaporations were performed in the 10^−8^ mbar range, with a current of 90 mA and 550 mA for the titanium and the platinum, respectively, while maintaining a constant deposition rate of 1 Å·s^−1^.

### 2.2. Structural and Electrical Characterization of ZnO Thin Films

The thickness of ZnO thin film samples was estimated on silicon substrates by ellipsometry (J. A. Woollam M2000 Ellipsometer) by measurements carried out for wavelengths between 300 nm and 1000 nm, with three different incident angles of 65°, 70° and 75°. X-ray diffractometry (XRD) (Diffractometer Bruker D8 Discover with Cu Kα radiation and a 5-axis Eulerian cradle) was conducted in a grazing incidence configuration (ω = 0.3°) to estimate the crystalline quality and the preferred crystalline orientation of the ZnO thin films deposited by ALD at various low temperatures deposition (i.e., between 60 °C and 100 °C), on different substrates (i.e., silicon, platinum and polyimide). The microstructure of the ZnO thin films was analysed by scanning electron microscopy (SEM) on a Helios 650 FIB-SEM instrument (FEI Technologies Inc., Hillsboro, OR, USA). Cross-sectional configurations were carried out to further confirm the thickness of the ZnO thin films measured by ellipsometry as well as its conformality on the polyimide and platinum substrates. The resistivity of the ZnO thin films was measured by the four-points probe technique on glass substrates using a sourcemeter (2400 Series SourceMeter, Keithley Instruments, Solon, OH, USA) coupled with a cylindrical four-point probe head and a probe station (Jandel Multiheight Probe Station, Jandel Engineering, Linslade, United Kingdom).

### 2.3. Strain Sensor and Equivalent Circuit Model

Schottky junctions were made from ALD growth of 300 nm thick ZnO thin films at a deposition temperature of 80 °C, on top of a polyimide substrate with 200 nm thick Pt IDE. The width and the spacing of the fingers of the comb electrodes were fixed to 10 µm. The bottom Pt IDE were patterned by a lift-off process while the ZnO thin films were selectively etched on top of the Pt electrodes by a FeCl_3_:H_2_O solution. The peculiar ALD features (e.g., low temperature processing, self-limiting nature and stoichiometric control at the nanoscale level) allow for a reliable microfabrication processing on flexible polymeric substrates. More details concerning the microfabrication process flow and the sensors’ electrical and transducing behaviour are available in our previous work [38].

**Figure 1:**
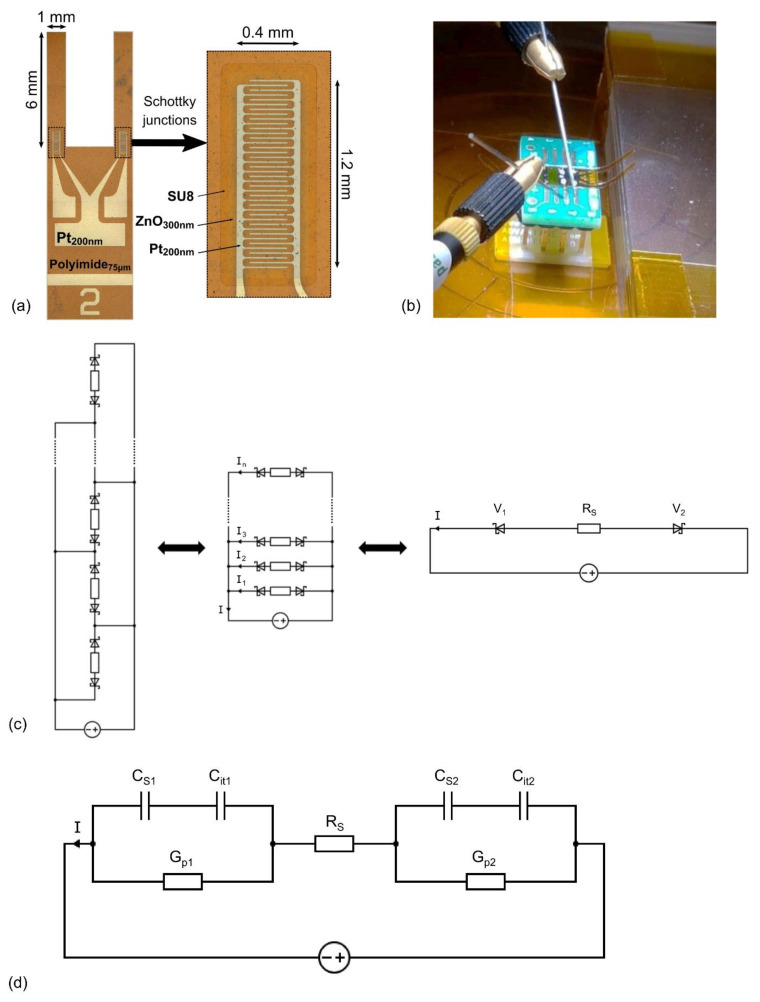
(**a**) Top view representation of a piezotronic strain microsensor. The black dashed line boxes represent a detailed view of the area of the interdigitated electrodes; (**b**) Piezotronic strain microsensor mounted and bonded on a printed circuit board (PCB), contacted with tungsten tips. The polyimide cantilevers are bent upwards, leading to the generation of a compressive strain; (**c**) Equivalent circuit model of the metal-semiconductor-metal structure with interdigitated electrodes; (**d**) Constitutive capacitance and conductance contributions from each Schottky diode.

As previously mentioned, the back-to-back Schottky diodes formed in our devices are defined by two sets of interdigitated platinum electrodes within the area defined by the ZnO pad, as shown in Figure 1a. The equivalent circuit model involving the interdigitated electrodes is presented in Figure 1c. This leads to the creation of several parallel back-to-back Schottky diodes arranged along the length of the cantilever. The total current I of the device corresponds to the sum of each individual back-to-back Schottky diode current, following the equation:

(1) $$I=I_{1}+I_{2}+I_{3}\;+\ldots +{\;I}_{n}=\overset{n}{\underset{i=1}{\sum }}I_{i}$$

where n corresponds to the number of back-to-back Schottky diodes created by the two sets of interdigitated platinum electrodes within the area defined by the ZnO pad. Since all the sets of back-to-back diodes were processed in a similar way, the current flowing out of every metal-semiconductor-metal (MSM) structure is considered to be equal ($I_{1}=I_{2}=I_{3}=\ldots ={\;I}_{n}$).

The equivalent circuit model can thus be represented by a single back-to-back Schottky diode corresponding to the sum of every MSM diode. This is linked to the use of the same planar metal bottom electrode layer, as well as to the uniformity of the ZnO coating and the reproducibility of its electrical properties by means of the ALD process. When a bias voltage is applied, either with a positive or negative value, one of the Schottky diode junctions will necessarily be reversely biased, while the other will be forward biased. In the following analysis, the reverse and forward biased Schottky junctions will be designated as 1 and 2, respectively. V_1_ and V_2_ are the voltage drops occurring at the reverse and forward biased Schottky diodes, respectively, while R_S_ represents the series resistance in the bulk of the ZnO thin film. Even if most of the voltage drop occurs at the reversely biased diode, a small voltage drop is necessary to bias the forward biased diode, as well as to take into account the series resistance of the semiconductor.

Equivalent circuit models of Schottky MS diodes are presented in Figure 1d, taking into account the different capacitive and conductive contributions from both Schottky MS diodes [41]. C_S_ is the capacitance corresponding to the Schottky depletion region, C_it_ is the capacitance induced by interface trap states, and G_p_ is the equivalent parallel conductance.

### 2.4. Electrical Characterization of the Pt/ZnO/Pt Schottky Junctions

**Figure 2:**
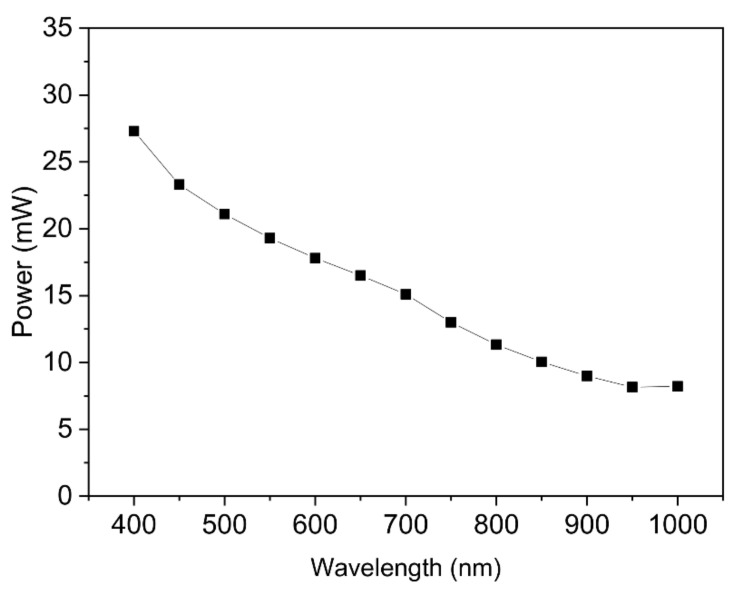
Characteristics of the microscope light between a wavelength of 400 nm and 1000 nm inducing light conditions during the electrical measurements. The Pt/ZnO/Pt cantilevered chip is placed at a distance of 10 cm from the light source.

A complete electrical characterization of the Pt/ZnO/Pt Schottky junctions was carried out by means of capacitance-voltage (C-V), capacitance-frequency (C-f), conductance-voltage (G-V) and conductance-frequency (G-f) measurements. These measurements were performed with an impedance analyser (E4990A Impedance Analyzer, Keysight Technologies, Santa Rosa, CA, USA). A constant AC modulation with an amplitude of 500 mV was superimposed on to a DC bias voltage swept over the defined tension range. The devices were contacted with tungsten tips of the PM8 probe station either completely in the dark, or under a microscope light (EasyLED Ringlights, SCHOTT North America Inc., Rye Brook, NY, USA) incident to the measured devices at a distance of 10 cm. The light characteristics were estimated using a photodiode (PM100D, Thorlabs, Newton, NJ, USA) and are presented in Figure 2. The junctions were let under bias voltage for 30 min either completely in the dark or under the microscope light to stabilize the output current value and to prevent signal drift before performing the (I-V) cycles.

The open and short calibration of the impedance analyser was realized prior to the measurements to remove the contribution of the cables and connections related to the device. Concerning the (C-V) and (G-V) measurements, the voltage was linearly swept between −10 V and 10 V, with a step voltage of 100 mV, for defined frequency values of 20 Hz, 60 Hz, 100 Hz, 500 Hz, 1 kHz, 10 kHz, 100 kHz and 1 MHz. Additional measurements were realized at lower frequencies where the above-mentioned NC phenomena are more prone to appear. Regarding the (C-f) and (G-f) measurements, the frequency was varied with a logarithmic sweep between 20 Hz and 1 MHz, for defined bias voltage values of 0 V, 500 mV, 1 V, 2 V, 4 V, 6 V, 8 V and 10 V. The (I-V) measurements were performed with an electrometer (6517B Electrometer/High Resistance Meter, Keithley Instruments, Solon, Ohio, USA) controlled by software (Labber, Lab Control Software Scandinavia AB, Vaxjo, Sweden). Additional (C-V) and (G-V) measurements were performed by bending the polyimide cantilevers upwards with a precise displacement, leading to the generation of controlled compressive strain steps calculated in the clamped area of the sensors. The upward bending of the polyimide cantilevers is shown in Figure 1b. The method of strain calculation is detailed in our previous work [38].

## 3. Results and Discussion

### 3.1. Structural and Electrical Properties of ZnO Thin Films

**Figure 3:**
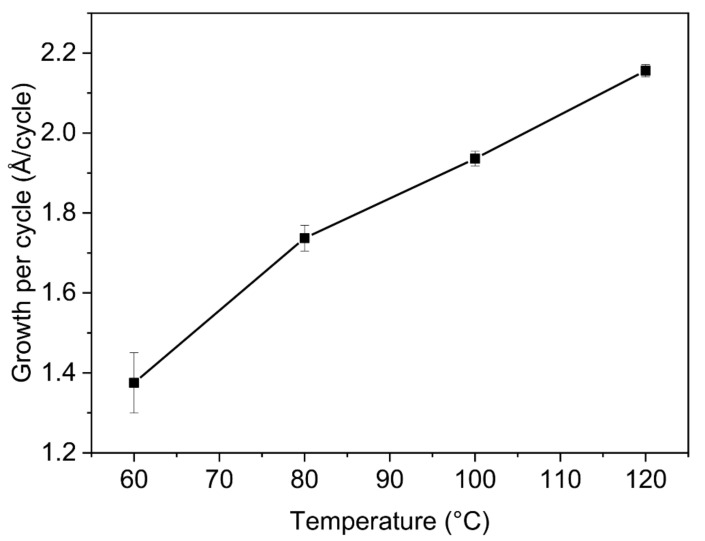
Growth rate per cycle (Å/cycle) of ZnO thin films by ALD for different deposition temperatures.

**Figure 4:**
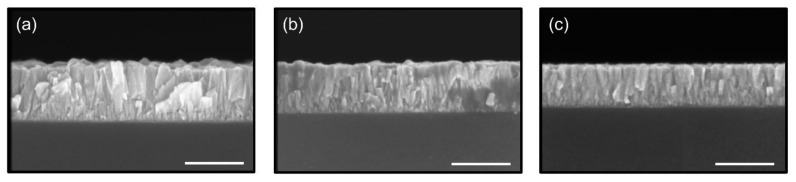
SEM cross-sectional images of ZnO thin films grown on Si substrates at (**a**) 100 °C, (**b**) 80 °C and (**c**) 60 °C. Each ZnO thin film was obtained with 1000 Atomic Layer Deposition (ALD) loops. The scale bar corresponds to 200 nm.

The growth rates per cycle (GPC) obtained for temperatures ranging from 60 °C to 120 °C, as well as the related cross-sectional micrographs depending on the growth temperature are illustrated on Figure 3 and Figure 4, respectively.

The GPC values obtained increase as deposition temperatures increase, indicating that the growth of ZnO thin films is outside the ALD temperature window for temperatures below 100 °C. This behaviour is typical of low reactivity reactions due to insufficient kinetic energy activation for the ligand exchange reactions, where low temperatures prevent complete reactions from occurring [42].

**Figure 5:**
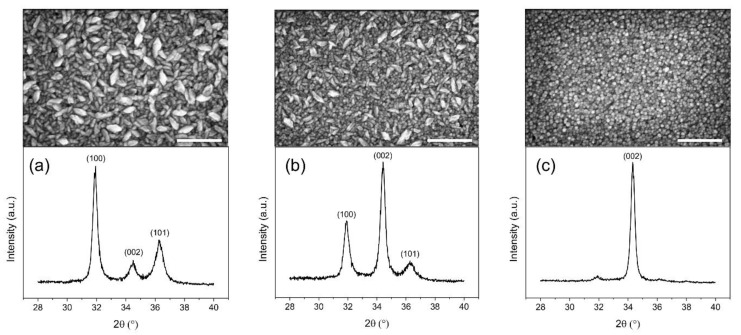
SEM top view images and associated GI-XRD diffraction patterns (ω = 0.3°) of ZnO thin films grown on Si substrates at a deposition temperature of (**a**) 100 °C, (**b**) 80 °C and (**c**) 60 °C. The obtained ZnO thin films were deposited with the same number of ALD loops (1000). The scale bar corresponds to 300 nm.

The SEM top view images of ZnO thin films grown by ALD on reference Si substrates with the associated grazing incident X-ray diffraction (GI-XRD) at a deposition temperature of (a) 100 °C, (b) 80 °C and (c) 60 °C are presented in Figure 5.

**Figure 6:**
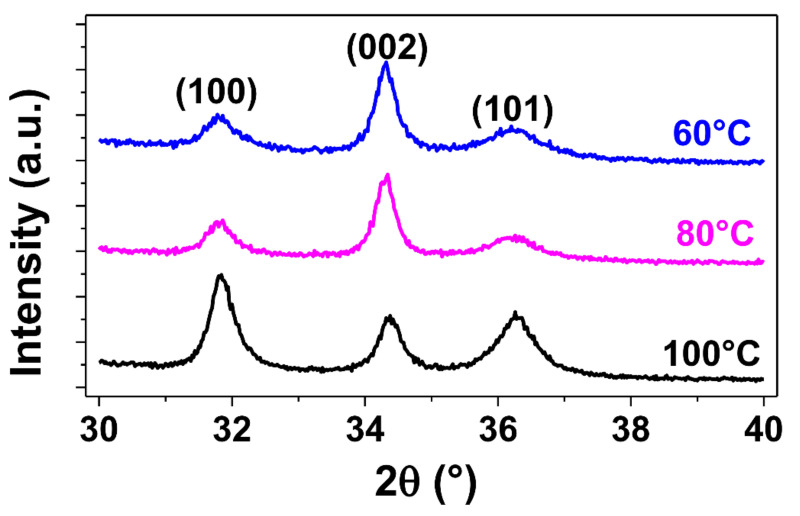
GI-XRD diffraction patterns (ω = 0.3°) of ZnO thin films grown on top of 75 µm thick polyimide substrates at 100 °C, 80 °C and 60 °C.

**Figure 7:**
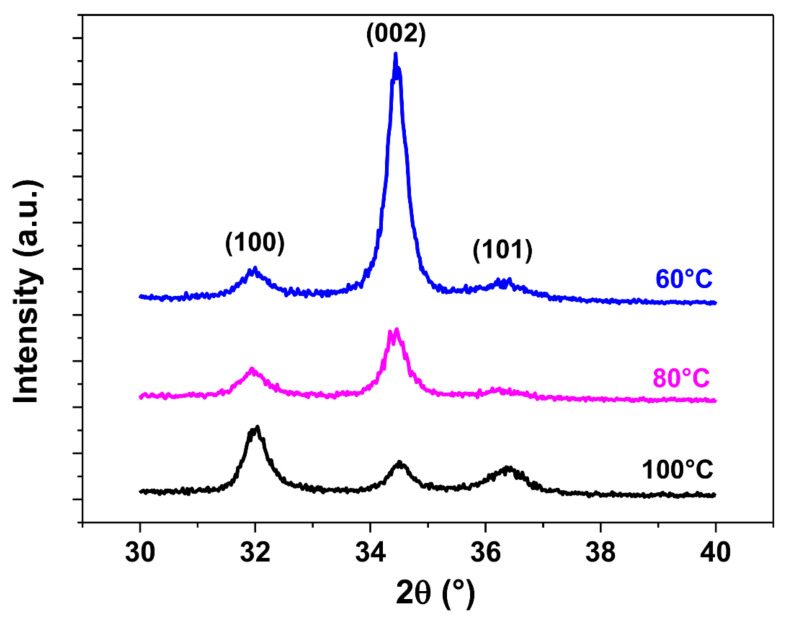
GI-XRD diffraction patterns (ω = 0.3°) of ZnO thin films grown on Si substrates coated with a 200 nm thick Pt layer at 100 °C, 80 °C and 60 °C.

The ZnO thin films deposited are polycrystalline. At a temperature of 100 °C, a different distribution of grain orientations can be observed, split between the (100), (002) and (101) crystalline orientations. This is further confirmed by SEM top view images showing a distribution of wedge-like shaped crystallites parallel to the substrate and of fine columnar crystallites perpendicular to the substrate at this temperature. However, a transition occurs as the deposition temperature decreases, with the (002) crystalline substantially increasing at 80 °C and becoming dominant at 60 °C. This is consistent with the appearance of fine columnar crystallites considerably increasing as deposition temperatures decrease. This results in a significant change in the morphology of the ZnO thin films obtained at lower temperatures, where grains are predominantly oriented in the (002) direction perpendicular to the substrate, along the c-axis, which is especially important for piezoelectric applications in order to maximize the collective piezoelectric participation of ZnO grains with a similar piezoelectric strain coefficient value and orientation [43,44,45]. The same observations can be applied to the growth of ZnO thin films on the sensors’ substrates, polyimide and platinum, as can be observed in Figure 6 and Figure 7, respectively.

**Figure 8:**
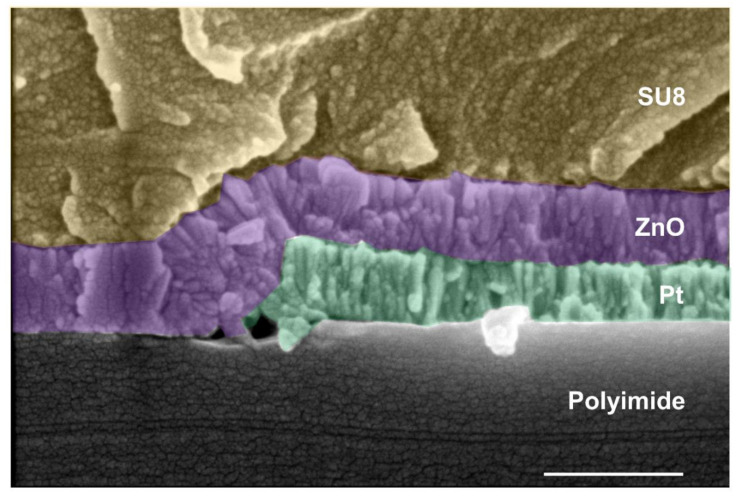
Cross-section showing the conformality of the ZnO thin film deposited by ALD on the polyimide substrate and the platinum metal electrodes. A SU8 resin top layer is deposited to protect the ZnO/Pt junction. The scale bar corresponds to 500 nm.

Furthermore, the conformality of the ALD technique is demonstrated in Figure 8, where a ZnO layer is deposited on a polyimide substrate as well as on the top of Pt electrodes. An encapsulation layer of SU8 resin is deposited on top to protect the ZnO/Pt junction against the environmental conditions and to maintain the electrical performance.

**Figure 9:**
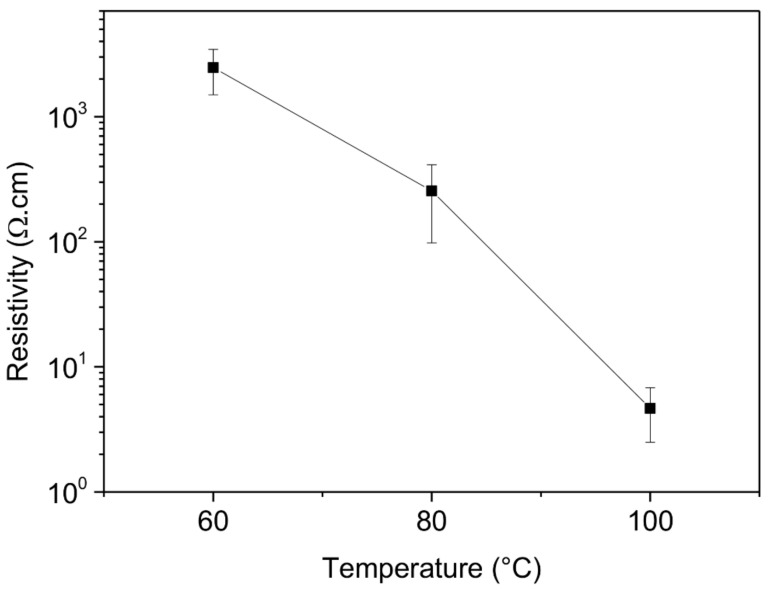
Evolution of the resistivity as a function of the deposition temperature for 150 nm thick ZnO thin films deposited at 60 °C, 80 °C and 100 °C on glass substrates, measured by the four-points probe method.

The resistivity of the ZnO thin films created was measured by the conventional four-points probe method on glass substrates [46]. The resistivity values obtained as a function of the deposition temperature are presented in Figure 9.

The resistivity increases from average values of 4.7 Ω·cm at 100 °C to 255.3 Ω·cm at 80 °C, reaching 2473.4 Ω·cm at 60 °C. These results confirm the increase of the resistivity for lower deposition temperatures by ALD. This last statement is well documented in the literature [47,48,49] and is linked to the reduction of ZnO defects (i.e., oxygen vacancies and zinc interstitials) at lower temperatures, as reported in our previous work [38]. This reduction in the defects contributes in turn to decrease the intrinsic n-type carrier concentration of the ZnO thin films at lower temperatures, within the aim of reaching appropriate values for a Schottky barrier formation with the Pt metal electrodes. The electrical and structural characteristics of the ZnO thin films deposited at 80 °C are thus well appropriated for their integration in MSM Schottky junctions.

### 3.2. Capacitance Modulation by Light

**Figure 10:**
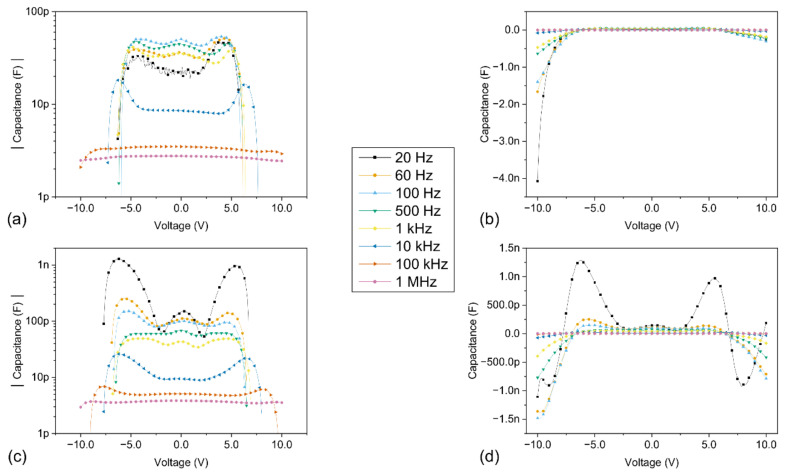
(C-V) characteristics under dark and light conditions, for different fixed frequencies of the AC modulation superimposed to the DC bias and ranging between 20 Hz and 1 MHz. The voltage was swept between −10 V and 10 V, with a step voltage of 100 mV; (**a**) log scale under dark conditions; (**b**) linear scale under dark conditions; (**c**) log scale under light conditions; (**d**) linear scale under light conditions. Only the positive capacitance values are displayed in the graphs (**a**,**c**) with a log scale.

**Figure 11:**
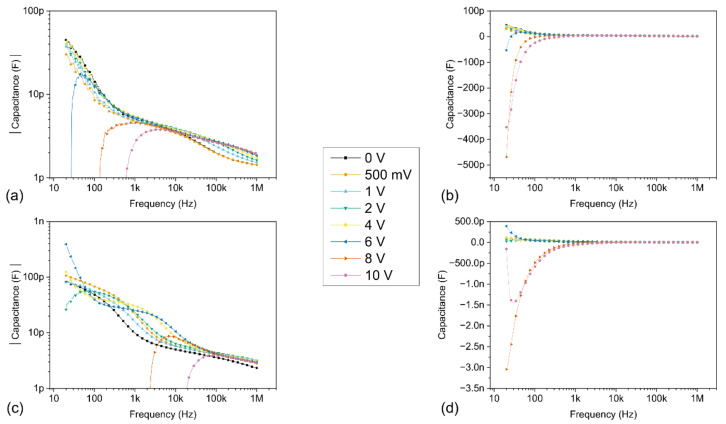
(C-f) characteristics under dark and light conditions for different fixed bias voltages ranging between 0 V and 10 V. The frequency was varied with a logarithmic sweep between 20 Hz and 1 MHz; (**a**) log scale under dark conditions; (**b**) linear scale under dark conditions; (**c**) log scale under light conditions; (**d**) linear scale under light conditions. Only the positive capacitance values are displayed in the graphs (**a**,**c**) with a log scale.

The capacitance-voltage (C-V) and capacitance-frequency (C-f) results of the Pt/ZnO/Pt Schottky junctions under the influence of dark and light conditions are presented in Figure 10 and Figure 11, respectively.

From Figure 10 and Figure 11, it can be clearly observed that the values of capacitance and conductance are dependent on both the frequency and the applied DC bias voltage. Most noticeably, the measured capacitance values at lower frequencies are very different from the Schottky junction capacitance values, which predict a gradual decrease of the capacitance as the bias voltage and the depletion region are increased [50]. This dispersive behaviour of the measured capacitance values as a function of the frequency depends on the ability of the charge carriers to follow the AC signal and is directly linked with the interface trap states at the forward biased Schottky junction. At lower frequencies, the charges localized at the interface trap states are able to follow the AC signal and yield an excess capacitance, corresponding to C_it_, whose value depends on the relaxation time of the interface trap states, and increases with decreasing frequencies. However, as the applied frequency is increased, the charges at the interface trap states are less and less able to follow the AC signal, which results in a decrease in the observed capacitance values, linked to the decrease of C_it_. The capacitance values are reduced to a few picoFarad at 1 MHz, effectively converging towards the value of the Schottky depletion region C_S_.

**Figure 12:**
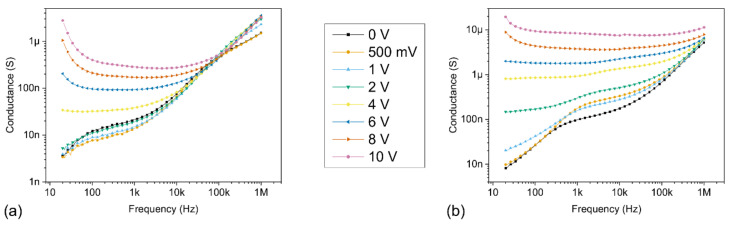
(G-f) characteristics under dark and light conditions for different fixed bias voltages ranging between 0 V and 10 V. The frequency was varied with a logarithmic sweep between 20 Hz and 1 MHz; (**a**) log scale under dark conditions; (**b**) log scale under light conditions.

Negative capacitance values are reported under the influence of both dark and light conditions, after reaching a threshold voltage value located above 5 V, which typically increases as the applied frequency is increased. Interestingly, no NC values are observed either for frequencies of 100 kHz and 1 MHz under dark conditions or for a frequency of 1 MHz under light conditions in the considered bias voltage range. The NC values are preceded by an increase in capacitance, localized in a peak with a positive capacitance value. Both the value and the broadening of this peak are significantly increased at lower frequencies, as well as in the presence of a light source. Furthermore, the positive capacitance values consistently increase in the presence of light over the entire bias voltage and frequency range explored. These results strongly suggest that both the presence of light and low frequencies favour the increase of the capacitance and the subsequent apparition of NC values in our devices, via the action of charges at the interface trap states. Additionally, the conductance-frequency (G-f) results under the influence of dark and light conditions are presented in Figure 12. These results reveal that the conductance values significantly increase in the presence of light over the entire bias voltage and frequency range explored.

**Figure 13:**
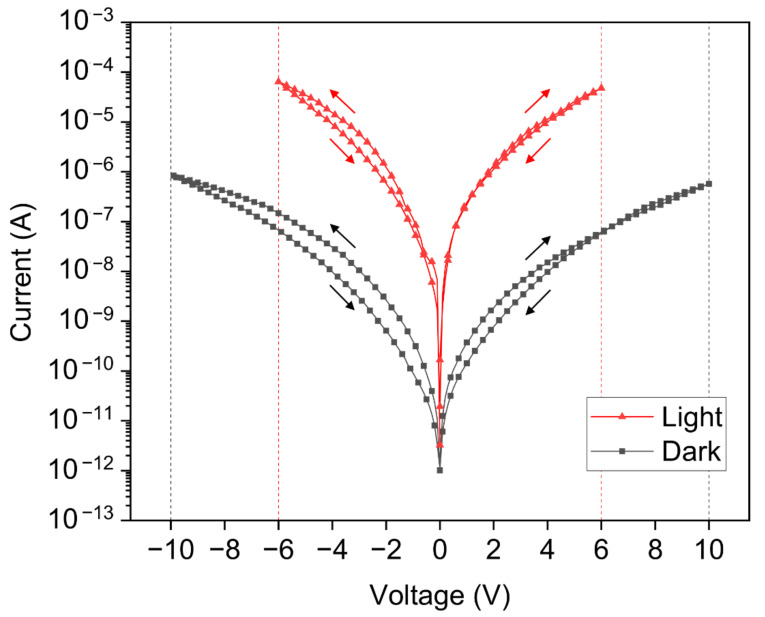
(I-V) characteristics under both dark and light conditions, measured with DC bias modulation. The current is represented on a logarithmic scale with absolute values. The current measurements were performed with an integration time of 200 ms, a step length of 100 mV, a sweep speed of 200 mV·s^−1^ and a delay of 300 ms between the step and the measurement. The voltage was swept for dark characteristics between −10 V and 10 V. The voltage was swept for light characteristics between −6 V and 6 V. The arrows indicate the parts of the curves corresponding to the forward and backward sweeps.

The light source generates electron-hole pairs in the bulk of the semiconductor, which actively contribute to the conduction mechanisms of the MSM diodes. This is further confirmed by the (I-V) characteristics of the devices shown in Figure 13, where the current values measured with a DC bias increase by over two orders of magnitude in the presence of light.

The hysteresis between the forward and backward sweep observed in the (I-V) curves in Figure 13 is linked to the filling of the interface trap states at the metal-semiconductor (MS) junctions. Indeed, during the forward sweeping (i.e., from zero bias to a higher bias voltage), the interface trap states are gradually filled up as the current and the bias voltage are increased. However, during the backward sweep (i.e., from a high bias voltage to zero bias), the initial high bias voltage is directly filling up the interface trap states. Some of these trap states remained filled up during the rest of the backward sweep to 0 V, which led to a lower density of interface trap states actively involved in the current conduction mechanism, reducing the current in the devices. This is linked to the different capture and re-emission charge carriers’ dynamics at the MS junctions, resulting in the hysteresis observed in the (I-V) curves. When the frequency is increased with an AC bias voltage modulation, the density of the interface trap states that respond is further reduced, leading to an increase in the hysteresis, as reported in our previous work [38]. A similar phenomenon can explain the discrepancy in the (C-V) characteristics presented in Figure 10 between the negative and positive bias voltage domains. Interestingly, the negative capacitance (NC) values reached higher values in the backward sweep than in the forward sweep. As the voltage is swept from a negative value up to a positive voltage value (i.e., from −10 V to 10 V), the different re-emission and capture charge carrier time constants are respectively involved in the negative and positive bias voltage regions of the (C-V) curves. As more charge carriers remain trapped during the re-emission process (i.e., from −10 V to zero bias), the capacitance values are slightly increased, especially in the presence of a light source.

As previously mentioned, the exact origin of the NC phenomena is still a matter of discussion in the literature. When a sufficiently large bias is applied, the drift component of the minority carriers (i.e., holes in our devices with n-type ZnO) becomes increasingly important, which can lead to an increase of their injection efficiency. However, the injection of minority carriers remains limited in wide bandgap semiconductors [41,51], such as zinc oxide with a bandgap of 3.3 eV [47]. Moreover, it is well known that the current transport in metal-semiconductor contacts is mainly due to majority carriers (i.e., electrons in our devices with n-type ZnO). Furthermore, a significant excess capacitance can be observed at lower frequencies even when small bias voltages are applied. Based on these considerations and the above-mentioned results, the NC phenomena observed have been linked with electrons that are captured and re-emitted at the interface trap states. The origin of NC values can be understood in terms of the variation of the interface charge density, by considering a continuous distribution of energy levels at the interface as proposed by Bardeen [52]. An increase in the capacitance values is thus linked to an increase in the interface charge density—or, in other words, in the electron capture process. In contrast, a decrease in the capacitance means that a loss of charges occurs at the interface, i.e., that electrons are being re-emitted or ejected from the interface trap states. Ultimately, negative capacitance values mean that the loss of electrons prevails over their capture at the MS interface. This simple model can be applied to explain the variation of the (C-V) curves in our devices for both dark and light conditions. As stated by Wu et al. [1], an impact-loss process has to be accounted for. When the electric field is increased, more and more hot electrons with higher energy are injected at the occupied interface trap states below the Fermi level, colliding with trapped electrons and creating empty states, which is linked to the loss of charges. Consequently, upon reaching a threshold voltage value, the capacitance values start to increase until they reach a peak of positive capacitance value. In this part of the curves, the electrons acquire enough energy to overcome the Schottky barrier and are captured at the interface trap states. As the bias voltage increases, more and more electrons are involved in the conduction mechanism. This results in an accumulation of charges at the interface, which leads to the increase of the capacitance values.

In the presence of a light source, supplementary electrons are photogenerated in the bulk of the semiconductor and injected at the MS interface, which significantly increases the accumulated charges at the interfaces, thus increasing the capacitance and its peak value. This is confirmed by the increase of both the capacitance and conductance values in the presence of light occurring at a lower bias voltage when compared to the dark characteristics. After reaching its peak value, the capacitance quickly decreases, indicating that the impact-loss process is growing, as electrons are being ejected from the interface trap states. Subsequently, negative capacitance values appear in the devices, meaning that the loss of electrons due to the impact-loss process exceeds the capture of electrons at the interface trap states. These phenomena are further marked at low frequencies, as more electrons are able to follow the AC signal through the interface trap states. A detailed comparison between the dark and light characteristics for every frequency and bias voltage in the study is provided in the Supplementary Materials, with Figures S1–S3 corresponding to the (C-V), (C-f) and (G-f) characteristics, respectively.

### 3.3. Capacitance Modulation by Light and Mechanical Strain

**Figure 14:**
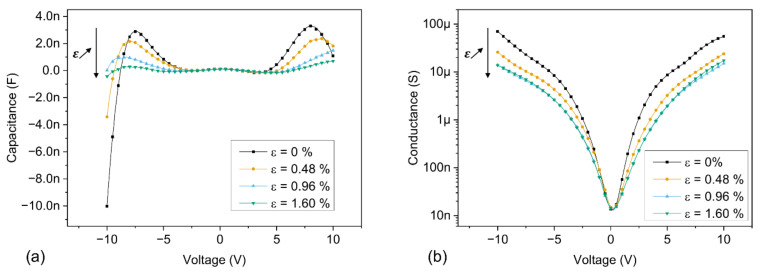
(C-V) and (G-V) characteristics under light conditions, for a fixed frequency of 20 Hz, with controlled compressive strain steps imposed on the junctions. The voltage was swept between −10 V and 10 V, with a step voltage of 100 mV; (**a**) (C-V) characteristics, linear scale; (**b**) (G-V) characteristics, log scale.

Additional capacitance and conductance were performed by taking advantage of the piezotronic nature of our devices. The upward bending of the polyimide cantilevers leads to the generation of controlled compressive strain steps at the clamped area of the piezotronic sensors, where the Pt/ZnO/Pt Schottky junctions are located. The strain induces the creation of localized piezoelectric polarization charges at the MS interfaces, which, in turn, effectively modulate the height of the Schottky barriers [53]. This has a direct impact on the electrical behaviour of the sensors by exponentially tuning the electrical current [38] and thus the flow of electrons passing through the MSM junctions. As the electrons are directly involved in the capacitive response of our devices at the MS interface, the capacitance is thus expected to be modulated with the strain accordingly. For a better visualization of the impact of the strain on the peak capacitance and on the NC values, measurements were performed at 20 Hz, after extended exposure to a light source. The results are presented in Figure 14.

Due to the application of a compressive strain, negative piezoelectric polarization charges are generated at the MS interfaces, increasing the height of the Schottky barrier. In turn, the electrical current is reduced, which reduces as well the flow of electrons passing through the MSM junctions. Consequently, the capacitance and conductance values are both reduced as the compressive strain imposed to the devices gradually increases, as can be seen in Figure 14. Most noticeably, the peak capacitance values decrease significantly as the strain is increased, while the apparition of NC values is delayed due to higher bias voltages. However, the impact of strain on the capacitance modulation becomes less and less significant both as the frequency increases and in dark conditions, as shown in Figure S4 in the Supplementary Materials. This simple method thus allows for an effective modulation of the capacitance by tuning the flow of electrons involved in the NC associated phenomena.

## 4. Conclusions

In conclusion, we report on the evidence of negative capacitance phenomena occurring at the interface of Schottky junctions made from the ALD growth of 300 nm thick ZnO thin films deposited on top of 200 nm thick Pt interdigitated electrodes. These metal-semiconductor-metal junctions are integrated at the clamped area of millimetre-sized polyimide cantilevers in piezotronic strain microsensors. The capacitance and conductance characteristics of the devices were studied over an extended bias voltage and frequency range, in the dark, as well as in the presence of a light source. We report on the observation of negative capacitance values over a wide frequency range, namely between 20 Hz and 1 MHz. The origin of the apparition of negative capacitance values has been linked to the injection of hot electrons due to an impact-loss process at the interface trap states of the metal-semiconductor junction. The NC values are preceded by a peak increase of the positive capacitance value, linked to the accumulation of trapped electrons at the interface trap states. These phenomena significantly increase at lower frequencies, as well as in the presence of a light source. Additionally, we propose an original way to modulate the capacitance and conductance characteristics by applying a mechanical strain to the devices, which effectively tunes the flow of electrons involved in the NC-associated phenomena. Thus, we propose a device that is simple to process by conventional microfabrication facilities and where the capacitance values can be modulated over a wide frequency range via the action of light and strain, while using easy-to-process materials. These results open up new perspectives and applications in the miniaturization of highly sensitive and low power consumption environmental sensors, as well as for broadband impedance matching by tank circuit in radio frequency applications. The inductance-like behaviour of capacitors with negative capacitance values is particularly interesting for the possibility of replacing bulky inductors that are challenging to process.

## Data Availability

The data that support the findings of this study are available from the corresponding author upon reasonable request.

## Supplementary Materials

The following are available online at https://www.mdpi.com/1424-8220/21/6/2253/s1, Figure S1: (C-V) characteristics under both dark and light conditions, for different fixed frequencies of the AC modulation superimposed on the DC bias and ranging between 20 Hz and 1 MHz. The voltage was swept between −10 V and 10 V, with a step voltage of 100 mV; (a) 20 Hz, log scale; (b) 20 Hz, linear scale; (c) 60 Hz, log scale; (d) 60 Hz, linear scale; (e) 100 Hz, log scale; (f) 100 Hz, linear scale; (g) 500 Hz, log scale; (h) 500 Hz, linear scale; (i) 1 kHz, log scale; (j) 1 kHz, linear scale; (k) 10 kHz, log scale; (l) 10 kHz, linear scale; (m) 100 kHz, log scale; (n) 100 kHz, linear scale; (o) 1 MHz, log scale; (p) 1 MHz, linear scale. Figure S2: (C-f) characteristics under both dark and light conditions for different fixed bias voltages ranging between 0 V and 10 V. The frequency was varied with a logarithmic sweep between 20 Hz and 1 MHz; (a) 0 V, log scale; (b) 0 V, linear scale; (c) 500 mV, log scale; (d) 500 mV, linear scale; (e) 1 V, log scale; (f) 1 V, linear scale; (g) 2 V, log scale; (h) 2 V, linear scale; (i) 4 V, log scale; (j) 4 V, linear scale; (k) 6 V, log scale; (l) 6 V, linear scale; (m) 8 V, log scale; (n) 8 V, linear scale; (o) 10 V, log scale; (p) 10 V, linear scale. Figure S3: (G-f) characteristics under both dark and light conditions for different fixed bias voltages ranging between 0 V and 10 V. The frequency was varied with a logarithmic sweep between 20 Hz and 1 MHz; (a) 0 V, log scale; (b) 500 mV, log scale; (c) 1 V, log scale; (d) 2 V, log scale; (e) 4 V, log scale; (f) 6 V, log scale; (g) 8 V, log scale; (h) 10 V, log scale. Figure S4: (C-V) characteristics under dark and light conditions for different fixed frequencies, with controlled compressive strain steps imposed on the junctions. The voltage was swept between −10 V and 10 V, with a step voltage of 100 mV; (a) dark conditions, 20 Hz; (b) under light conditions, 20 Hz; (c) dark conditions, 1 kHz; (d) under light conditions, 1 kHz; (e) dark conditions, 1 MHz; (f) under light conditions, 1 MHz.
