# Supplementary material for: Evidence of Negative Capacitance and Capacitance Modulation by Light and Mechanical Stimuli in Pt/ZnO/Pt Schottky Junctions
Source: Sensors (Basel). 2021 Mar 23;21(6):2253. doi: 10.3390/s21062253 (PMC8004606; doi:10.3390/s21062253)
Supplement: Supplementary file 1 [file sensors-21-02253-s001.pdf]

Supplementary Materials

Evidence of Negative Capacitance and Capacitance Modulation by Light and Mechanical Stimuli in Pt/ZnO/Pt Schottky Junctions

Raoul Joly[1,2], Stéphanie Girod[1], Noureddine Adjeroud[1], Patrick Grysan[1] and Jérôme Polesel-Maris[1,*]

[1] Luxembourg Institute of Science and Technology, L-4422 Belvaux, Luxembourg; raoul.joly@list.lu (R.J.); stephanie.girod@list.lu (S.G.); noureddine.adjeroud@list.lu (N.A.); patrick.grysan@list.lu (P.G.)
[2] Limpertsberg Campus, University of Luxembourg, 162a Avenue de la Faïencerie, L-1511 Luxembourg, Luxembourg
* Correspondence: jerome.polesel@list.lu

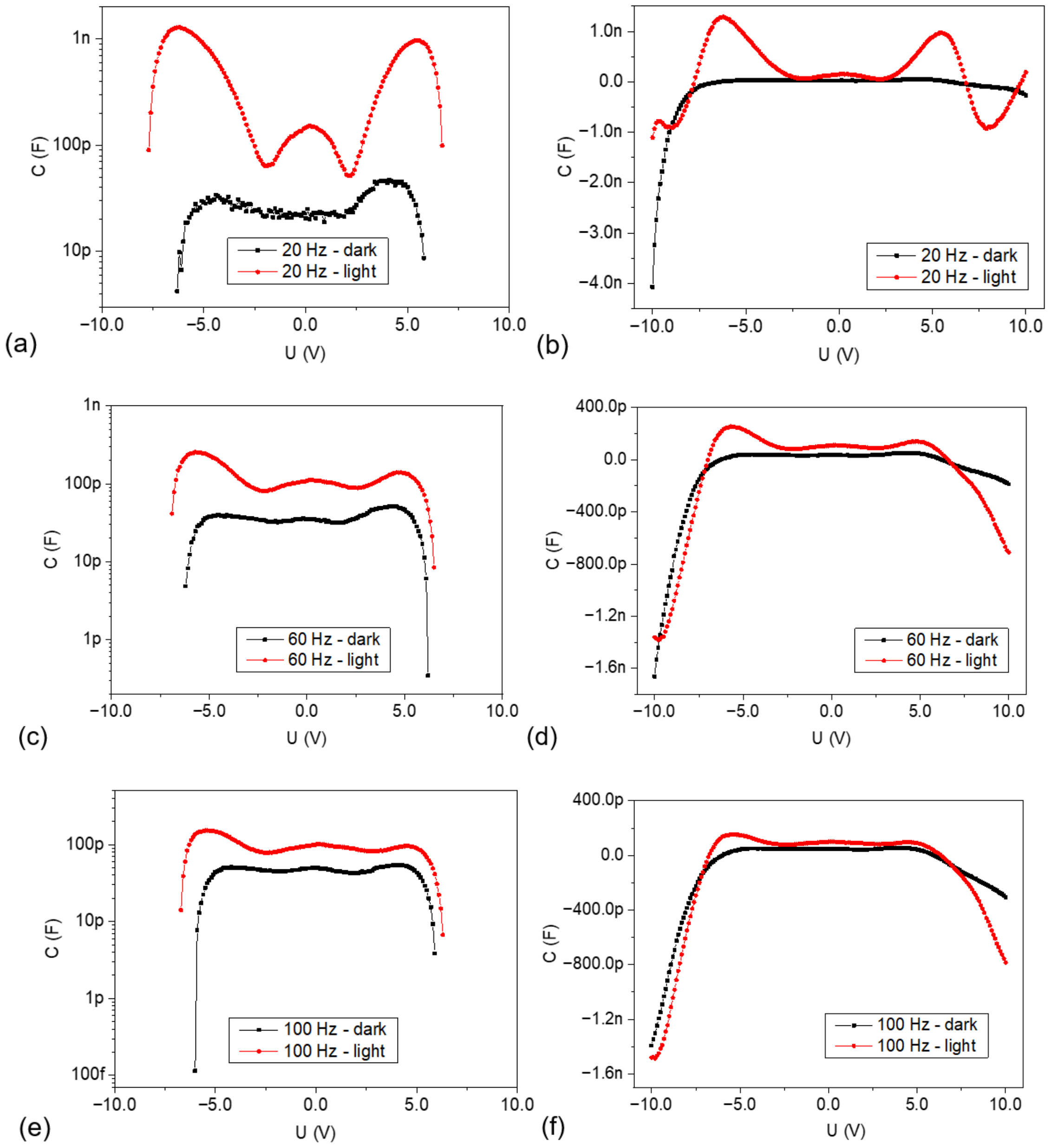

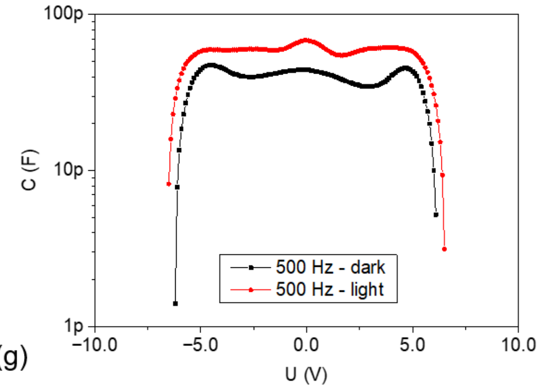
100p
10p
1p
C (F)
−10.0
−5.0
0.0
5.0
10.0
U (V)
500 Hz - dark
500 Hz - light

(g)

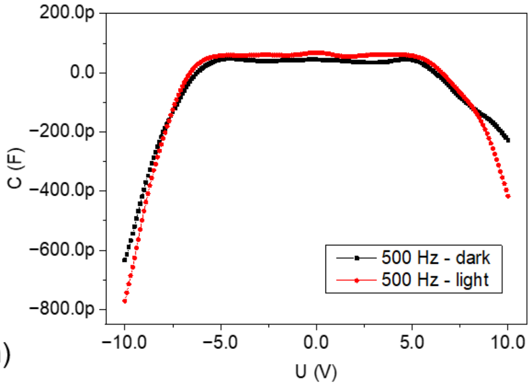
200.0p
0.0
−200.0p
−400.0p
−600.0p
−800.0p
C (F)
−10.0
−5.0
0.0
5.0
10.0
U (V)
500 Hz - dark
500 Hz - light

(h)

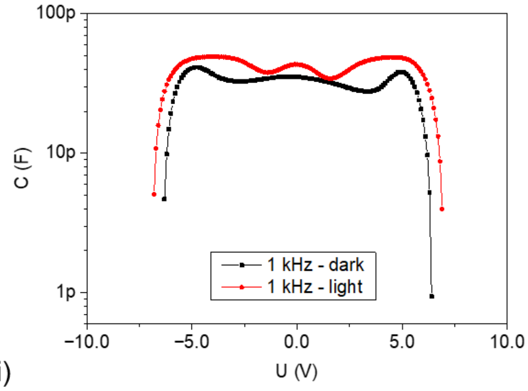
100p
10p
1p
C (F)
−10.0
−5.0
0.0
5.0
10.0
U (V)
1 kHz - dark
1 kHz - light

(i)

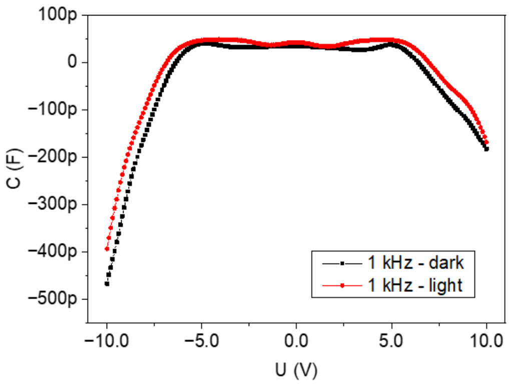
100p
0
−100p
−200p
−300p
−400p
−500p
C (F)
−10.0
−5.0
0.0
5.0
10.0
U (V)
1 kHz - dark
1 kHz - light

(j)

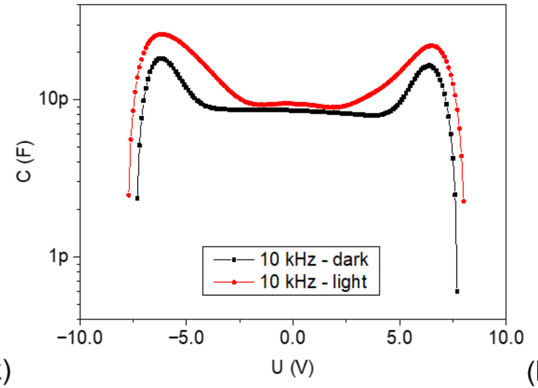
10p
1p
C (F)
−10.0
−5.0
0.0
5.0
10.0
U (V)
10 kHz - dark
10 kHz - light

(k)

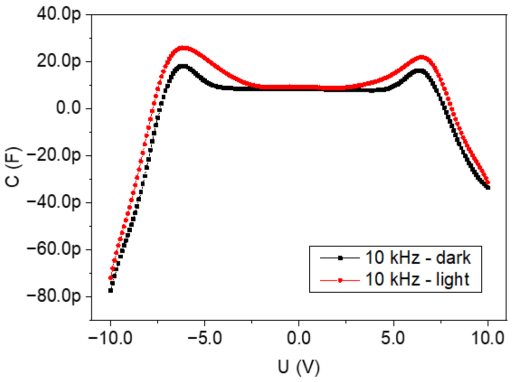
40.0p
20.0p
0.0
−20.0p
−40.0p
−60.0p
−80.0p
C (F)
−10.0
−5.0
0.0
5.0
10.0
U (V)
10 kHz - dark
10 kHz - light

(l)

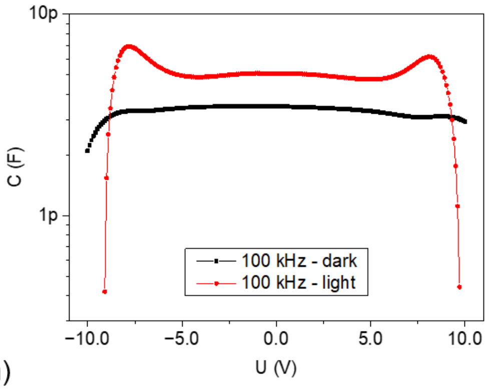
10p
1p
C (F)
−10.0
−5.0
0.0
5.0
10.0
U (V)
100 kHz - dark
100 kHz - light

(m)

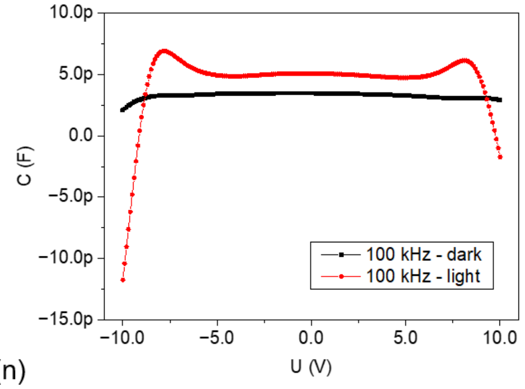
10.0p
5.0p
0.0
−5.0p
−10.0p
−15.0p
C (F)
−10.0
−5.0
0.0
5.0
10.0
U (V)
100 kHz - dark
100 kHz - light

(n)

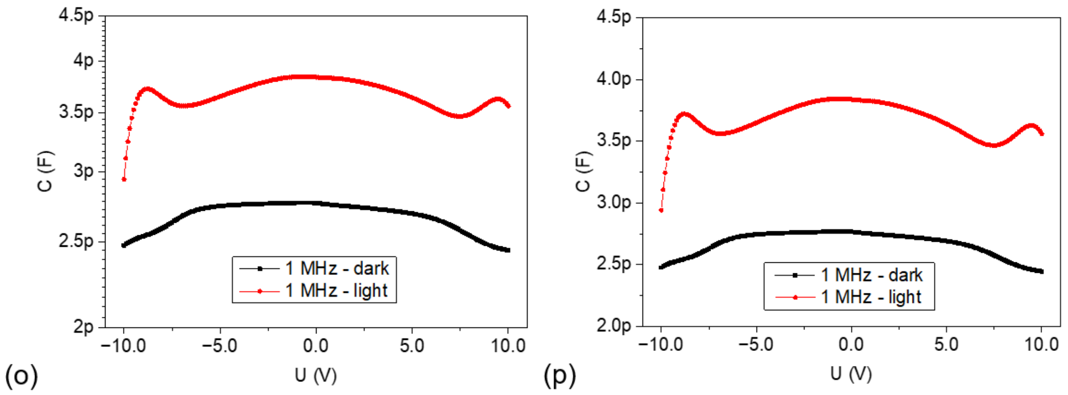

Figure S1. (C-V) characteristics under both dark and light conditions, for different fixed frequencies of the AC modulation superimposed to the DC bias and ranging between 20 Hz and 1 MHz. The voltage was swept between -10 V and 10 V, with a step voltage of 100 mV; (**a**) 20 Hz, log scale; (**b**) 20 Hz, linear scale; (**c**) 60 Hz, log scale; (**d**) 60 Hz, linear scale; (**e**) 100 Hz, log scale; (**f**) 100 Hz, linear scale; (**g**) 500 Hz, log scale; (**h**) 500 Hz, linear scale; (**i**) 1 kHz, log scale; (**j**) 1 kHz, linear scale; (**k**) 10 kHz, log scale; (**l**) 10 kHz, linear scale; (**m**) 100 kHz, log scale; (**n**) 100 kHz, linear scale; (**o**) 1 MHz, log scale; (**p**) 1 MHz, linear scale.

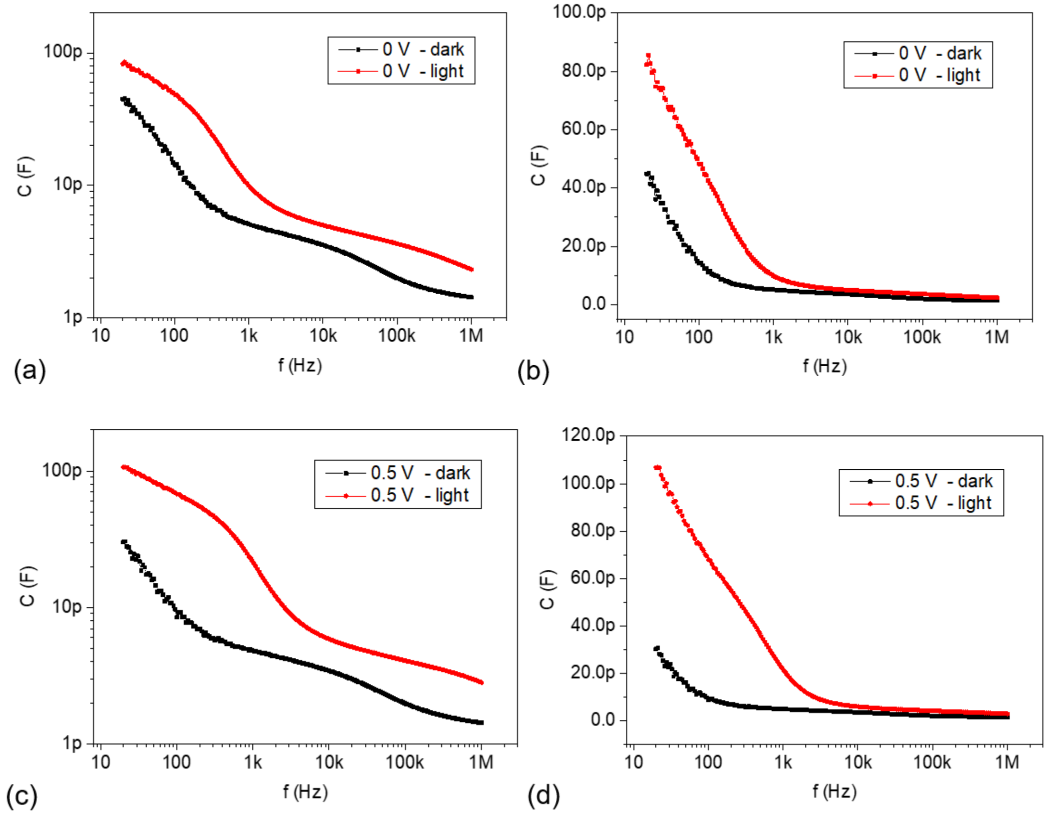

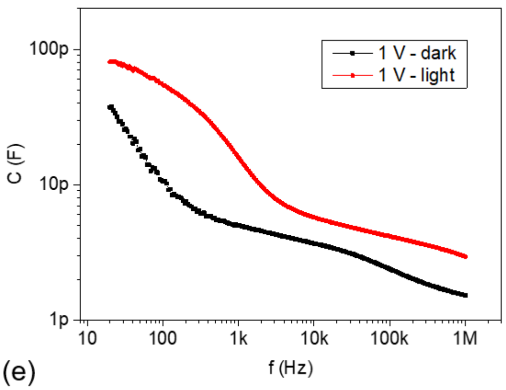
1 V - dark
1 V - light
C (F)
f (Hz)

(e)

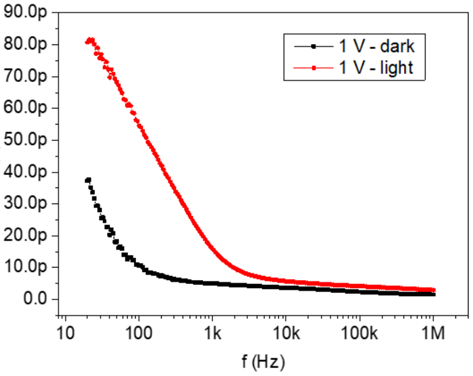
1 V - dark
1 V - light
C (F)
f (Hz)

(f)

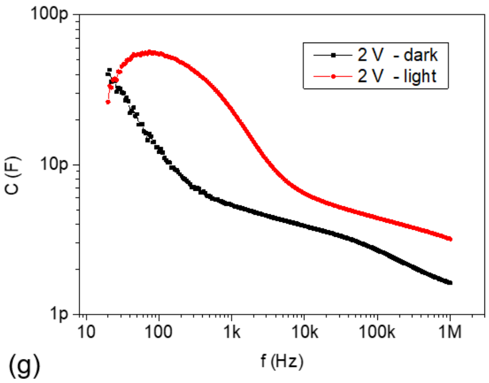
2 V - dark
2 V - light
C (F)
f (Hz)

(g)

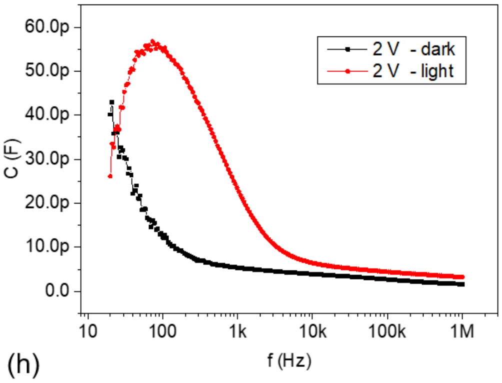
2 V - dark
2 V - light
C (F)
f (Hz)

(h)

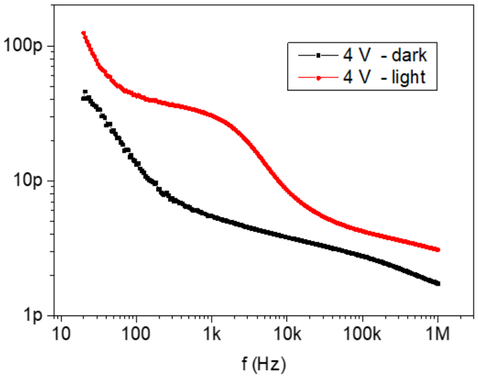
4 V - dark
4 V - light
C (F)
f (Hz)

(i)

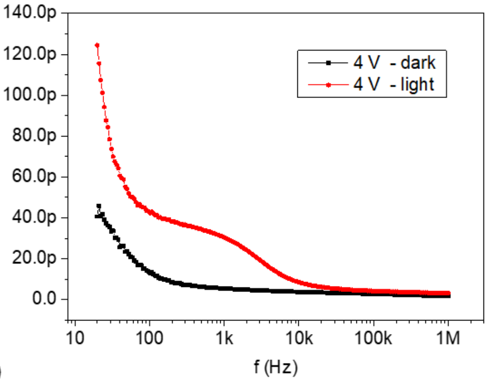
4 V - dark
4 V - light
C (F)
f (Hz)

(j)

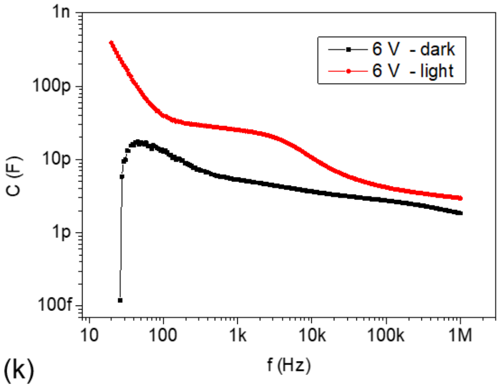
6 V - dark
6 V - light
C (F)
f (Hz)

(k)

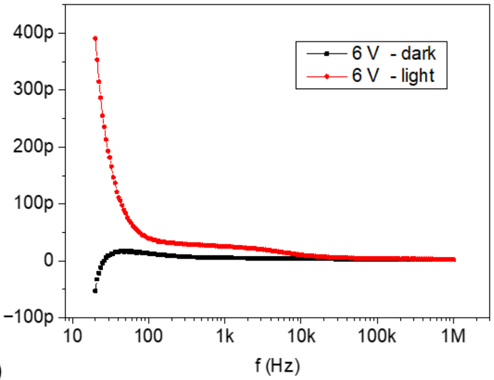
6 V - dark
6 V - light
C (F)
f (Hz)

(l)

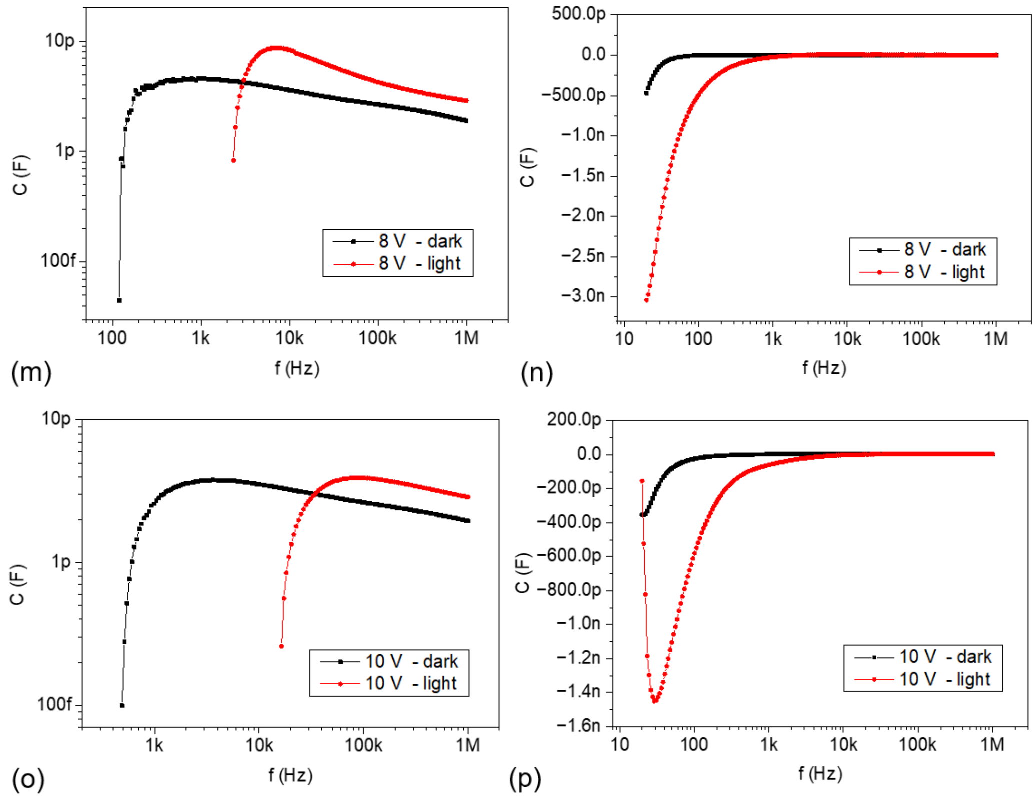

Figure S2. (C-f) characteristics under both dark and light conditions for different fixed bias voltages ranging between 0 V and 10 V. The frequency was varied with a logarithmic sweep between 20 Hz and 1 MHz; (**a**) 0 V, log scale; (**b**) 0 V, linear scale; (**c**) 500 mV, log scale; (**d**) 500 mV, linear scale; (**e**) 1 V, log scale; (**f**) 1 V, linear scale; (**g**) 2 V, log scale; (**h**) 2 V, linear scale; (**i**) 4 V, log scale; (**j**) 4 V, linear scale; (**k**) 6 V, log scale; (**l**) 6 V, linear scale; (**m**) 8 V, log scale; (**n**) 8 V, linear scale; (**o**) 10 V, log scale; (**p**) 10 V, linear scale.

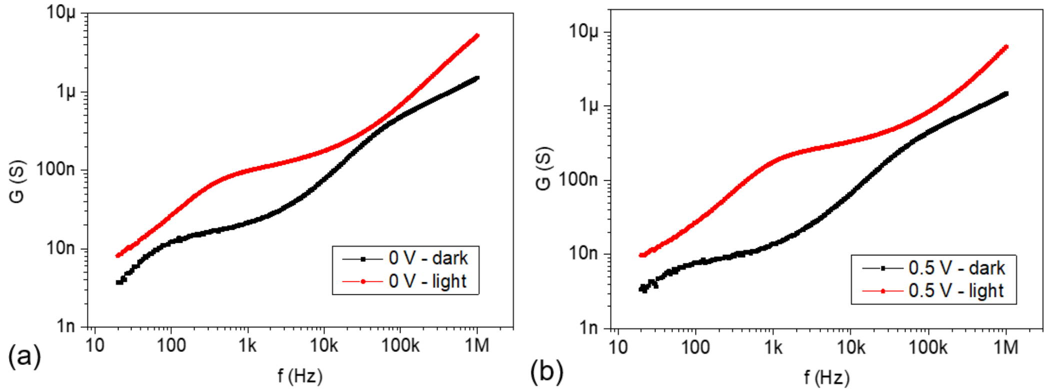

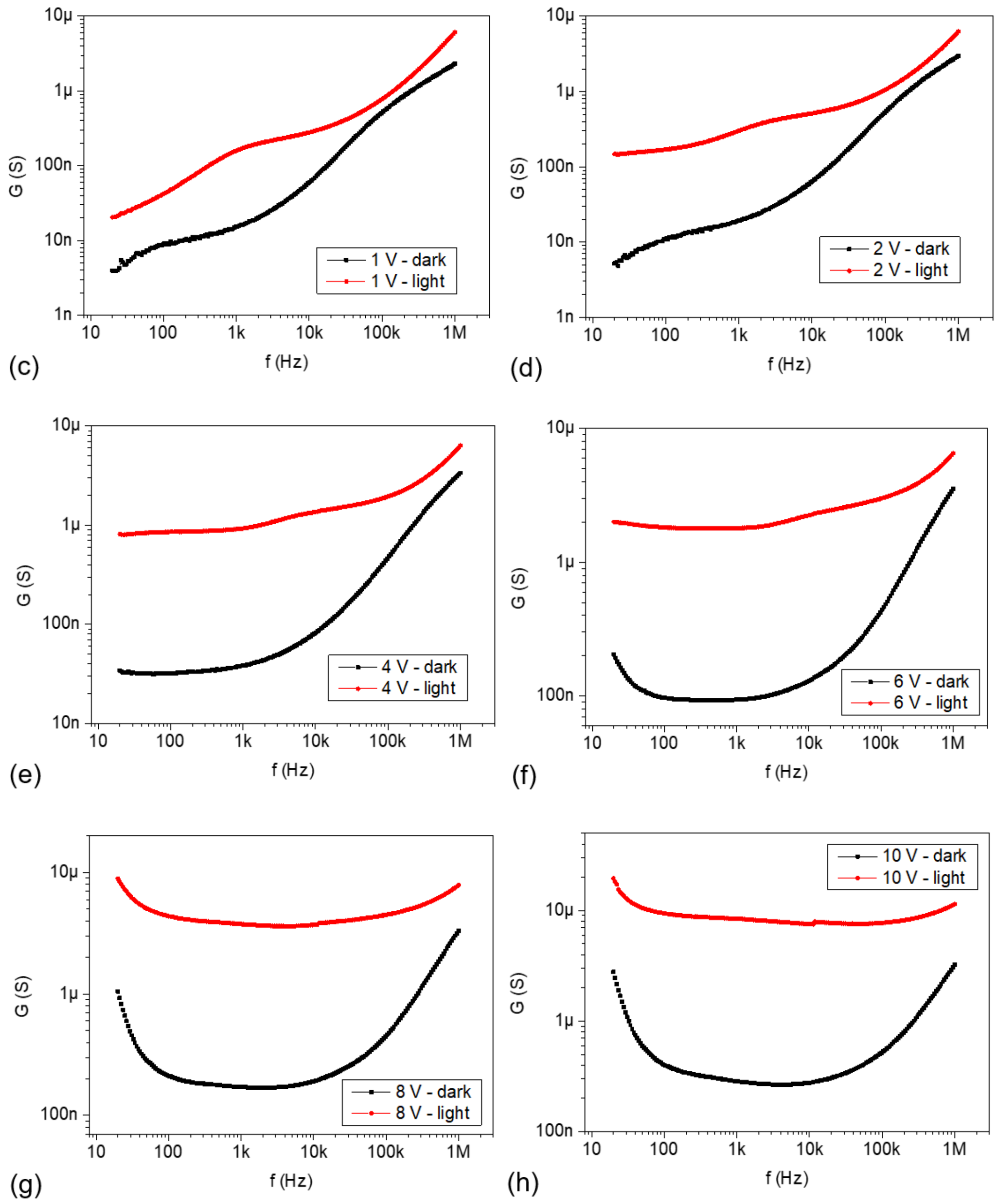

Figure S3. (G-f) characteristics under both dark and light conditions for different fixed bias voltages ranging between 0 V and 10 V. The frequency was varied with a logarithmic sweep between 20 Hz and 1 MHz; (**a**) 0 V, log scale; (**b**) 500 mV, log scale; (**c**) 1 V, log scale; (**d**) 2 V, log scale; (**e**) 4 V, log scale; (**f**) 6 V, log scale; (**g**) 8 V, log scale; (**h**) 10 V, log scale.

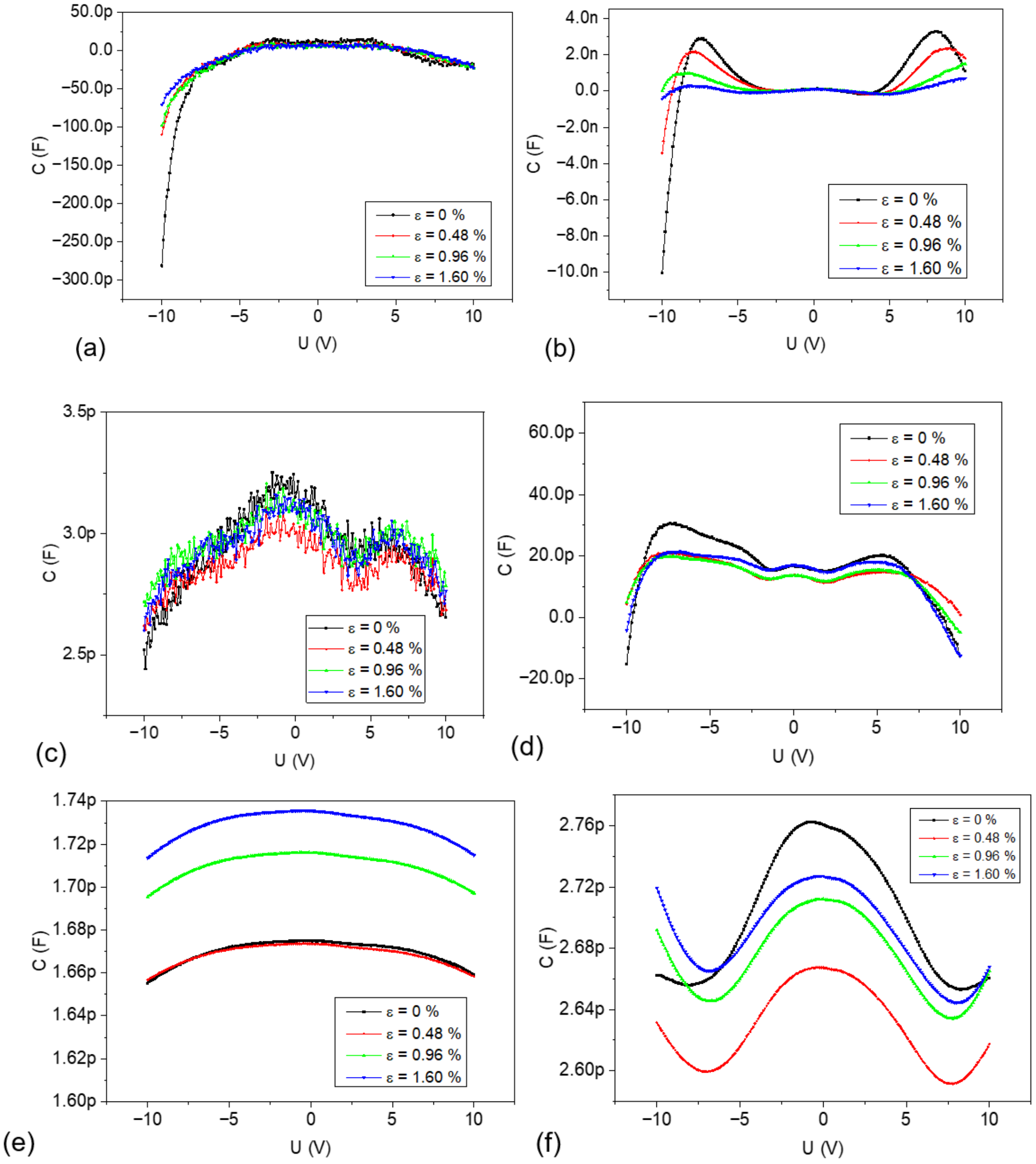

Figure S4. (C-V) characteristics under dark and light conditions for different fixed frequencies, with controlled compressive strain steps imposed on the junctions. The voltage was swept between −10 V and 10 V, with a step voltage of 100 mV; (**a**) dark conditions, 20 Hz; (**b**) light conditions, 20 Hz; (**c**) dark conditions, 1 kHz; (**d**) light conditions, 1 kHz; (**e**) dark conditions, 1 MHz; (**f**) light conditions, 1 MHz.
